# Improved spatial speckle contrast model for tissue blood flow imaging: effects of spatial correlation among neighboring camera pixels

**DOI:** 10.1117/1.JBO.28.12.125002

**Published:** 2023-12-05

**Authors:** Julio Cesar Juarez-Ramirez, Beatriz Coyotl-Ocelotl, Bernard Choi, Ruben Ramos-Garcia, Teresita Spezzia-Mazzocco, Julio C. Ramirez-San-Juan

**Affiliations:** a Instituto Nacional de Astrofisica, Optica y Electronica, Departamento de Optica, Tonantzintla, Mexico; b University of California, Irvine, Beckman Laser Institute and Medical Clinic, Department of Surgery, Irvine, California, United States

**Keywords:** laser speckle contrast imaging, correlation, blood flow

## Abstract

**Significance:**

Speckle contrast analysis is the basis of laser speckle imaging (LSI), a simple, inexpensive, noninvasive technique used in various fields of medicine and engineering. A common application of LSI is the measurement of tissue blood flow. Accurate measurement of speckle contrast is essential to correctly measure blood flow. Variables, such as speckle grain size and camera pixel size, affect the speckle pattern and thus the speckle contrast.

**Aim:**

We studied the effects of spatial correlation among adjacent camera pixels on the resulting speckle contrast values.

**Approach:**

We derived a model that accounts for the potential correlation of intensity values in the common experimental situation where the speckle grain size is larger than the camera pixel size. *In vitro* phantom experiments were performed to test the model.

**Results:**

Our spatial correlation model predicts that speckle contrast first increases, then decreases as the speckle grain size increases relative to the pixel size. This decreasing trend opposes what is observed with a standard speckle contrast model that does not consider spatial correlation. Experimental data are in good agreement with the predictions of our spatial correlation model.

**Conclusions:**

We present a spatial correlation model that provides a more accurate measurement of speckle contrast, which should lead to improved accuracy in tissue blood flow measurements. The associated correlation factors only need to be calculated once, and open-source software is provided to assist with the calculation.

## 1 Introduction

A speckle pattern is created when coherent light is reflected from a rough surface or transmitted through a scattering media. This pattern is made up of regions with high and low intensity and depends on the distribution of scatterers. Static scatterers create a static speckle pattern, while dynamic scatterers create a dynamic speckle pattern. Speckle patterns appear in images collected with many detection systems, including radar,^1^^,^^2^ microscopy,^3^^–^^5^ astronomy,^6^^–^^8^ and ultrasound.^9^^,^^10^ Biological samples produce speckle patterns with different dynamics in different regions, such as those associated with blood vessels. Regions rich in blood vessels, like skin, are of particular interest to monitor skin lesions, such as wounds, ulcers, and tumors. It has also been used to assess the efficacy of various treatments, such as topical creams and laser therapy. Additionally, speckle pattern imaging can be used to study the effects of aging and diseases, such as diabetes, on skin microcirculation. When recorded by a camera with finite exposure time, dynamic speckle patterns are blurred, and this local blurring is related to the movement of scatterers, such as blood flow, and can be measured as speckle contrast (K).

Speckle contrast analysis is the basis of laser speckle contrast imaging (LSCI), a low-cost, noninvasive technique used in different areas of medicine, such as dentistry,^11^^,^^12^ ophthalmology,^13^^,^^14^ dermatology,^15^^,^^16^ neurobiology,^17^^,^^18^ and neuroscience.^19^^,^^20^ Common uses of LSCI are to measure blood flow^21^^,^^22^ and perform intraoperative assessment of tissue perfusion.^23^

For LSCI, the propagation of coherent light through scattering media produces a speckle pattern captured with a digital camera. If the medium contains moving scatterers, the speckle pattern fluctuates proportionally to the scatterers’ speed. If the exposure time is longer than the correlation time of the backscattered light, the speckle visibility will be reduced. In 1981, Fercher and Briers first measured blood flow speed using LSCI^24^ and derived the following expression for the contrast K:

$$K=\frac{\sigma }{\left\langle I\right\rangle }={\left(\frac{\tau_{c}}{2T}\{1-e^{-\frac{2T}{\tau_{c}}}\}\right)}^{1/2},$$ (1)

where T is the camera exposure time, $\tau_{c}$ is the correlation time of the backscattered light from the sample, σ is the standard deviation and ⟨I⟩ the average intensity.

Since this seminal paper, the LSCI technique has evolved and therefore the contrast calculation too. For example, Lemieux and Durian^25^ added a correction factor β (which depends on the ratio of the pixel and speckle grain size) to the Siegert relation that links the intensity dynamics associated with scatterer motion with the electric field dynamics,^26^ resulting in $g_{2}(\tau )=1+\beta {\left|g_{1}(\tau )\right|}^{2}$, where $g_{1}(\tau )$ is the electric field autocorrelation function and $g_{2}(\tau )$ is the intensity autocorrelation function. Bandyopadhyay et al.^27^ corrected an over simplification on the derivation of Eq. (1), applying a double integral of $\tau =t_{1}-t_{2}$, obtaining

$$K^{2}=\beta \frac{e^{-2x}-1+2x}{2x^{2}},$$ (2)

where $x=T/\tau_{c}$. This expression still considers only the light scattered from dynamic scatterers and dimensionless pixels. Several groups^19^^,^^25^^,^^27^^,^^28^ have since demonstrated the necessity of considering speckle not only from dynamic scatterers but also stationary scatterers. Static scatterers affect the Siegert relation^25^ and thus the contrast, resulting in a revised form of $g_{2}$:

$$g_{2}(\tau )=1+A\beta {\left|g_{1}(\tau )\right|}^{2}+B\beta |g_{1}(\tau )|,$$ (3)

where $A=\frac{I_{f}^{2}}{(I_{f}+I_{s}{)}^{2}}$, $B=\frac{2I_{f}I_{s}}{(I_{f}+I_{s}{)}^{2}}$, and $I_{s}$ and $I_{f}$ represent the contributions from the static and dynamical scattered light, respectively:

$$K=\beta^{1/2}{\left[\rho^{2}\frac{e^{-2x}-1+2x}{2x^{2}}+4\rho (1-\rho )\frac{e^{-x}-1+x}{x^{2}}+(1-\rho {)}^{2}\right]}^{1/2}+C_{n},$$ (4)

where $\rho =\frac{I_{f}}{\left(I_{f}+I_{s}\right)}$ is the fraction of the fluctuating component of light that interacts with dynamic scatterers, and $C_{n}$ is an offset term that considers the noise contribution to the measurement.

Bandyopadhyay et al.^27^ proposed a technique to measure β experimentally. Accounting for the finite size of the camera pixels^29^ led to an analytical expression for β, assuming Gaussian illumination:

$$\beta^{1/2}(M)=\sqrt{1/M}\;\mathrm{erf}(\sqrt{\pi M})-(1/(\pi M))(1-e^{-\pi M}),$$ (5)

where M is the ratio between the pixel area and the speckle area (i.e., the speckle grain size). $\beta^{1/2}(M)$ effectively corresponds to the spatial contrast with a sliding window of one pixel size. Equation (5) describes the effect of pixel and speckle sizes on the theoretical calculation of speckle contrast. However, it does not consider the likely correlation among neighboring pixels.

With the derivation of the equations above, the correlation between neighboring pixels is neglected, and hence are valid only for small speckle area compared to the pixel area (M≥1). When the speckle area is larger than the pixel area (M<1), these models do not fit experimental data well. Here we present a mathematical model that considers correlations between neighboring pixels to improve the agreement between theoretical and experimental contrast measurements for any size of speckle and pixel areas (M>0).

## 2 Theory

### 2.1 Spatial Correlation Model

Several computational algorithms^30^^,^^31^ exist to calculate the speckle contrast from a single raw speckle image or set of images. Application of spatial statistics^24^^,^^32^ to a single image is probably the most common approach. This analysis typically implements a sliding square window to calculate the local speckle contrast as the standard deviation of pixel intensities divided by the mean intensity. However, the process of the sliding window is not taken into in the current theoretical model (any of the above mentioned models), which can lead to an inaccurate contrast calculation, and therefore to an inaccurate blood flow calculation. Indeed, it is necessary to consider not only the contribution of the physical size of the camera’s pixel (autocorrelation) but also the effect of the size of the sliding window to improve the accuracy of β and therefore of the contrast.

Skipetrov et al.^33^ employed a sliding window of 3×3 pixels. In this work, we expand on their analysis to extend it to a sliding window of any size to measure an accurate blood flow speed. We show that the mathematical model considering the statistical analysis of the sliding window agrees well with experimental data.

**Fig. 1:**
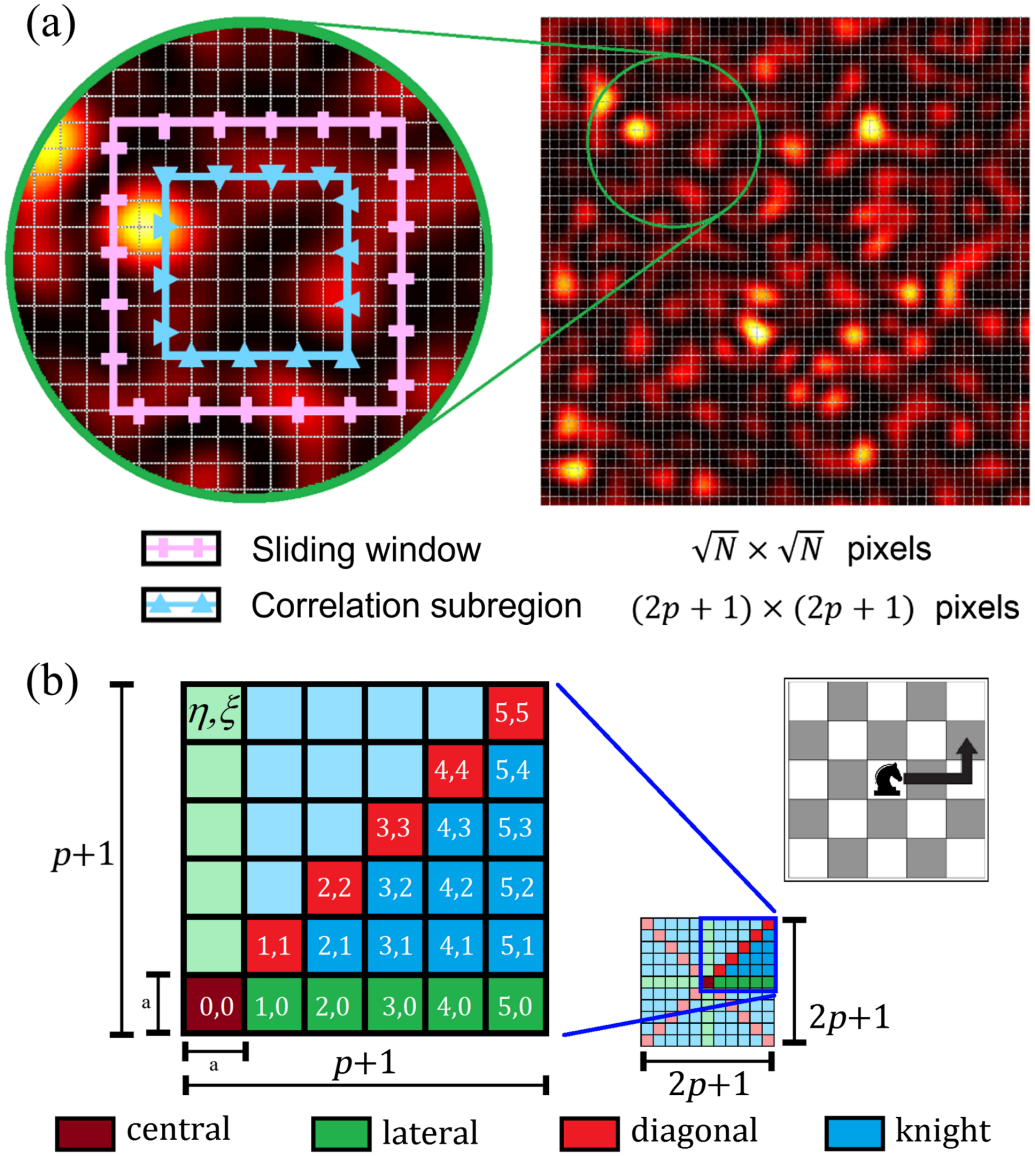
(a) Schematic representation of the sliding window of ($\sqrt{N}\times \sqrt{N}$) pixels (pink) and the correlation subregion of $(2p+1{)}^{2}$ (blue). In this example, the sliding window of size $\sqrt{N}=11$, and correlation subregion of size 2p+1=7 is shown. (b) Schematic representation of the correlation region of $(2p+1{)}^{2}$ pixels, with a close-up view of the first quadrant, and a chess board showing the movement of a knight. Some pixels are labeled with their coordinates, and they are colored according to their type (see text for details).

We assume a speckle pattern $I(\vec{r})$ recorded by a digital camera, where r→=xi^+yj^ is the position vector on the CCD image. Here we consider a sliding window of $\sqrt{N}\times \sqrt{N}$ pixels, with N being an odd number. We define the speckle spot size as comprising a subregion of (2p+1)×(2p+1) pixels in size, where $p\in Z^{+}$ and $2p+1\leq \sqrt{N}$. Within this subregion, the pixels are assumed to be spatially correlated. Figure 1(a) shows a schematic representation of the sliding window (pink rectangles) and one possible example of a correlation subregion (blue triangles).

Figure 1(b) shows the corresponding coordinates (η,ξ) for each pixel of the sliding window, where $\eta ,\xi \in \{0,\pm 1,\pm 2\ldots ,\pm \frac{\sqrt{N}-1}{2}\}$ and a correlation subregion containing different types of correlations: central, lateral, diagonal, and knight. It is important to mention that all pixels within the subregion are correlated and all of them are considered on calculations. However, due to symmetry, many of the correlations are repeated several times. For example, since the lateral pixels (0,η), (η,0), (0,−η), and (−η,0) are all at the same distance from the central pixel of the sliding window, they are equally correlated with the central pixel, and therefore, this correlation value is repeated four times.

Considering a pixel with area $a^{2}$, the analytic expression for the speckle contrast calculated over an arbitrary sliding window size of $\sqrt{N}\times \sqrt{N}$ pixels, and hence an arbitrary correlation subregion of (2p+1)×(2p+1) pixels may be written as (details can be found in Appendix):

$$K_{s}^{2}(N,p)=\frac{1}{\mu_{0,0}}-\frac{1}{N(N-1)}(4\overset{p}{\underset{\eta =1}{\sum }}(\sqrt{N}-\eta )\sqrt{N}\frac{1}{\mu_{\eta ,0}}+4\overset{p}{\underset{\eta =1}{\sum }}{\left(\sqrt{N}-\eta \right)}^{2}\frac{1}{\mu_{\eta ,\eta }}\;+8\overset{p-1}{\underset{\xi =1}{\sum }}\overset{p}{\underset{\eta =\xi +1}{\sum }}(\sqrt{N}-\eta )(\sqrt{N}-\xi )\frac{1}{\mu_{\eta ,\xi }}),$$ (6)

where each summation inside the parentheses stands for a diagonal, lateral, or knight-type correlation, respectively. As described in Appendix, Eq. (6) is valid for p≥2, i.e., a 5×5 subregion. For p=1, there is no knight correlation, and thus the double summation does not exist. For p=0, there is no lateral, diagonal, or knight-type correlation, and thus the terms in parentheses equal zero. Here we consider only p≥2. $1/\mu_{\eta ,\xi }$ is the correlation factor of the pixel with coordinates (η,ξ) and with the central pixel (0,0) of the sliding window, given by

$$\frac{1}{\mu_{\eta ,\xi }}=\frac{1}{a^{4}}\int_{a(2\xi -1)/2}^{a(2\xi +1)/2}\int_{a(2\eta -1)/2}^{a(2\eta +1)/2}\int_{-a/2}^{a/2}\int_{-a/2}^{a/2}g_{1}^{2}(x-x^{\prime },y-y^{\prime })dx\;dy\;dx^{\prime }\;dy^{\prime },$$ (7)

where $g_{1}$ is a first-order correlation function. At this point, we have not considered the correlation function $\left(g_{1}^{2}(\overset{\to }{r}-{\overset{\to }{r}}^{\prime })\right)$; this will be the topic of the next section. The factor β in Eq. (4) corresponds to the spatial contrast $K_{s}^{2}(N,p)$ (i.e., $\beta =K_{s}^{2}(N,p)$) for any window size, while Eq. (5), $K_{s}^{2}(N,p)$ takes into account not only the finite dimension of the pixel but its possible spatial correlation.

Table 1 shows expressions for the spatial contrast using Eq. (6) for the first four different dimensions of the sliding window.

**Table 1:** Expressions for Eq. (6) for sliding window sizes of 1×1 (i.e., autocorrelation), 3×3, 5×5, and 7×7  pixels.

$K_{s}^{2}(1^{2},0)$	$\frac{1}{\mu_{0,0}}$
$K_{s}^{2}(3^{2},1)$	$\frac{1}{\mu_{0,0}}+(-\frac{1}{3}\frac{1}{\mu_{1,0}})+(-\frac{2}{9}\frac{1}{\mu_{1,1}})$
$K_{s}^{2}(5^{2},2)$	$\frac{1}{\mu_{0,0}}+(-\frac{2}{15}\frac{1}{\mu_{1,0}}-\frac{1}{10}\frac{1}{\mu_{2,0}})+(-\frac{8}{75}\frac{1}{\mu_{1,1}}-\frac{3}{50}\frac{1}{\mu_{2,2}})+(-\frac{4}{25}\frac{1}{\mu_{2,1}})$
$K_{s}^{2}(7^{2},3)$	$\frac{1}{\mu_{0,0}}+(-\frac{1}{14}\frac{1}{\mu_{1,0}}-\frac{5}{84}\frac{1}{\mu_{2,0}}-\frac{1}{21}\frac{1}{\mu_{3,0}})+(-\frac{3}{49}\frac{1}{\mu_{1,1}}-\frac{25}{588}\frac{1}{\mu_{2,2}}-\frac{4}{147}\frac{1}{\mu_{3,3}})+(-\frac{5}{49}\frac{1}{\mu_{2,1}}-\frac{4}{49}\frac{1}{\mu_{3,1}}-\frac{10}{147}\frac{1}{\mu_{3,2}})$

As it seen in Table 1, the contribution of each lateral, diagonal, and knight correlation decreases with increasing subregion size.

### 2.2 Gaussian Velocity Distribution Function

For a Gaussian-shaped laser beam, the spatial correlation function also is Gaussian in shape.

#### 2.2.1 Analytical solutions

The Gaussian correlation function $g_{1}^{2}(\overset{\to }{r}-{\overset{\to }{r}}^{\prime })=\exp[-\frac{\pi }{2}\frac{{\left(x-x^{\prime }\right)}^{2}+{\left(y-y^{\prime }\right)}^{2}}{A_{c}}]$^33^^,^^34^ may be written using a change of variables $w=x-x^{\prime }$, $\overset{¯}{w}=(x+x^{\prime })/2$, $u=y-y^{\prime }$, and $\overset{¯}{u}=(y+y^{\prime })/2$:

$$g_{1}(w,u)=\exp[-\frac{\pi }{2}\frac{\left(w^{2}+u^{2}\right)}{A_{c}}],$$ (8)

where $A_{c}$ is the correlation area (i.e., speckle size). For a speckle area with radius b:

$$g_{1}^{2}(w,u)={\left(\exp[-\frac{\pi }{2}\frac{\left(w^{2}+u^{2}\right)}{\pi b^{2}}]\right)}^{2}=\exp[-(w^{2}+u^{2})/b^{2}].$$ (9)

Substituting Eq. (9) into Eq. (7), we obtain

$$\frac{1}{\mu_{\eta ,\xi }}=\frac{1}{4\pi^{2}M^{2}}\times (e^{-\pi M(\eta -1{)}^{2}}-2e^{-\pi M\eta^{2}}+e^{-\pi M(\eta +1{)}^{2}}+\pi \sqrt{M}((\eta -1)\mathrm{erf}[\sqrt{\pi M}(\eta -1)]-2\eta \mathrm{erf}[\sqrt{\pi M}\eta ]+(\eta +1)\mathrm{erf}[\sqrt{\pi M}(\eta +1)]))\times (e^{-\pi M{\left(\xi -1\right)}^{2}}-2e^{-\pi M\xi^{2}}+e^{-\pi M{\left(\xi +1\right)}^{2}}+\pi \sqrt{M}((\xi -1)\mathrm{erf}[\sqrt{\pi M}(\xi -1)]-2\xi \mathrm{erf}(\sqrt{\pi M}\xi )+(\xi +1)\mathrm{erf}[\sqrt{\pi M}(\xi +1)])),$$ (10)

where $M=a^{2}/\pi b^{2}$ is the ratio of the pixel area and the speckle area. Equation (10) provides an expression for speckle contrast that is similar to that described in section 4.6 of Ref. 35.

Equations (6) and (10) are programmed and available in MATLAB and Mathematica (see Code and Data Availability).

One particular case is when p=0 (i.e., autocorrelation), corresponding to a spatial correlation subregion of only a single pixel. For this case, we obtain the well-established analytic expression for spatial speckle contrast:^29^

$$K_{s}^{2}(N,p=0)=\frac{1}{\mu_{0,0}}=\frac{1}{\pi^{2}M^{2}}{\left(-1+e^{-\pi M}+\pi \sqrt{M}\mathrm{erf}[\sqrt{\pi M}]\right)}^{2}.$$ (11)

Equation (11) is identical to the correction factor β [see Eq. (5)] proposed in Refs. 19 and 25.

Table 2 shows the first $1/\mu_{\eta ,\xi }$ factors needed to calculate the speckle contrast for 1×1, 3×3, 5×5, and 7×7 sliding windows.

**Table 2:** Correlation factors calculated using Eq. (10) needed to calculate the speckle contrast of a 7×7 sliding window.

Central	
$\frac{1}{\mu_{0,0}}$	$\frac{1}{M^{2}\pi^{2}}{\left(-1+e^{-M\pi }+\sqrt{M}\pi \mathrm{erf}(\sqrt{M}\sqrt{\pi })\right)}^{2}$
Lateral	
$\frac{1}{\mu_{1,0}}$	$\frac{1}{2M^{2}\pi^{2}}(-1+e^{-M\pi }+\sqrt{M}\pi \mathrm{erf}(\sqrt{M}\sqrt{\pi }))(1+e^{-4M\pi }-2e^{-M\pi }\;-2\sqrt{M}\pi (\mathrm{erf}(\sqrt{M}\sqrt{\pi })-\mathrm{erf}(2\sqrt{M}\sqrt{\pi })))$
$\frac{1}{\mu_{2,0}}$	$\frac{1}{2M^{2}\pi^{2}}(-1+e^{-M\pi }+\sqrt{M}\pi \mathrm{erf}(\sqrt{M}\sqrt{\pi }))(e^{-9M\pi }-2e^{-4M\pi }+e^{-M\pi }\;+\sqrt{M}\pi (\mathrm{erf}(\sqrt{M}\sqrt{\pi })-4\mathrm{erf}(2\sqrt{M}\sqrt{\pi })+3\mathrm{erf}(3\sqrt{M}\sqrt{\pi })))$
$\frac{1}{\mu_{3,0}}$	$\frac{1}{2M^{2}\pi^{2}}(-1+e^{-M\pi }+\sqrt{M}\pi \mathrm{erf}(\sqrt{M}\sqrt{\pi }))(e^{-16M\pi }-2e^{-9M\pi }+e^{-4M\pi }\;+2\sqrt{M}\pi (\mathrm{erf}(2\sqrt{M}\sqrt{\pi })-3\mathrm{erf}(3\sqrt{M}\sqrt{\pi })+2\mathrm{erf}(4\sqrt{M}\sqrt{\pi })))$
Diagonal	
$\frac{1}{\mu_{1,1}}$	$\frac{1}{4M^{2}\pi^{2}}(1+e^{-4M\pi }-2e^{-M\pi }-2\sqrt{M}\pi {\left(\mathrm{erf}(\sqrt{M}\sqrt{\pi })-\mathrm{erf}(2\sqrt{M}\sqrt{\pi }))\right)}^{2}$
$\frac{1}{\mu_{2,2}}$	$\frac{1}{4M^{2}\pi^{2}}(e^{-9M\pi }-2e^{-4M\pi }+e^{-M\pi }+\sqrt{M}\pi {\left(\mathrm{erf}(\sqrt{M}\sqrt{\pi })-4\mathrm{erf}(2\sqrt{M}\sqrt{\pi })+3\mathrm{erf}(3\sqrt{M}\sqrt{\pi }))\right)}^{2}$
$\frac{1}{\mu_{3,3}}$	$\frac{1}{4M^{2}\pi^{2}}(e^{-16M\pi }-2e^{-9M\pi }+e^{-4M\pi }+2\sqrt{M}\pi (\mathrm{erf}(2\sqrt{M}\sqrt{\pi })-3\mathrm{erf}(3\sqrt{M}\sqrt{\pi })+2\mathrm{erf}(4\sqrt{M}\sqrt{\pi })){)}^{2}$
Knight	
$\frac{1}{\mu_{2,1}}$	$\frac{1}{4M^{2}\pi^{2}}(1+e^{-4M\pi }-2e^{-M\pi }-2\sqrt{M}\pi (\mathrm{erf}(\sqrt{M}\sqrt{\pi })-\mathrm{erf}(2\sqrt{M}\sqrt{\pi })))(e^{-9M\pi }-2e^{-4M\pi }+e^{-M\pi }\;+\sqrt{M}\pi (\mathrm{erf}(\sqrt{M}\sqrt{\pi })-4\mathrm{erf}(2\sqrt{M}\sqrt{\pi })+3\mathrm{erf}(3\sqrt{M}\sqrt{\pi })))$
$\frac{1}{\mu_{3,1}}$	$\frac{1}{4M^{2}\pi^{2}}(1+e^{-4M\pi }-2e^{-M\pi }-2\sqrt{M}\pi (\mathrm{erf}(\sqrt{M}\sqrt{\pi })-\mathrm{erf}(2\sqrt{M}\sqrt{\pi })))(e^{-16M\pi }-2e^{-9M\pi }+e^{-4M\pi }\;+2\sqrt{M}\pi (\mathrm{erf}(2\sqrt{M}\sqrt{\pi })-3\mathrm{erf}(3\sqrt{M}\sqrt{\pi })+2\mathrm{erf}(4\sqrt{M}\sqrt{\pi })))$
$\frac{1}{\mu_{3,2}}$	$\frac{1}{4M^{2}\pi^{2}}(e^{-9M\pi }-2e^{-4M\pi }+e^{-M\pi }+\sqrt{M}\pi (\mathrm{erf}(\sqrt{M}\sqrt{\pi })-4\mathrm{erf}(2\sqrt{M}\sqrt{\pi })\;+3\mathrm{erf}(3\sqrt{M}\sqrt{\pi })))(e^{-16M\pi }-2e^{-9M\pi }+e^{-4M\pi }\;+2\sqrt{M}\pi (\mathrm{erf}(2\sqrt{M}\sqrt{\pi })-3\mathrm{erf}(3\sqrt{M}\sqrt{\pi })+2\mathrm{erf}(4\sqrt{M}\sqrt{\pi })))$

#### 2.2.2 Numerical solution

**Fig. 2:**
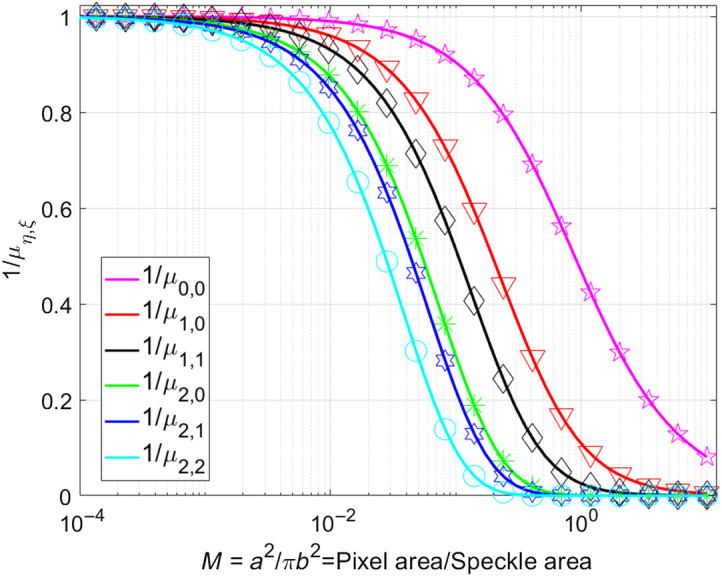
Comparison between the analytical expression (solid lines) using Eq. (10) and a numerical solution (symbols) of Eq. (7) using Mathematica 11.

We integrated Eq. (7) numerically using Mathematica 11 and compared the resultant solution to the analytical solution in Eq. (10). A good agreement between the analytic and numerical solution is observed (Fig. 2).

The parameters $1/\mu_{0,0}$, $1/\mu_{1,0}$, and $1/\mu_{1,1}$ are sufficient to calculate the contrast K for a spatial correlation subregion of 3×3  pixels.^36^ For a subregion of 5×5  pixels, we also should include the terms $1/\mu_{2,0}$, $1/\mu_{2,1}$, and $1/\mu_{2,2}$.^37^ Now it is possible to calculate the speckle contrast using Eq. (10) for any arbitrary odd size of sliding window ($\sqrt{N}$) and subregion (2p+1).

**Fig. 3:**
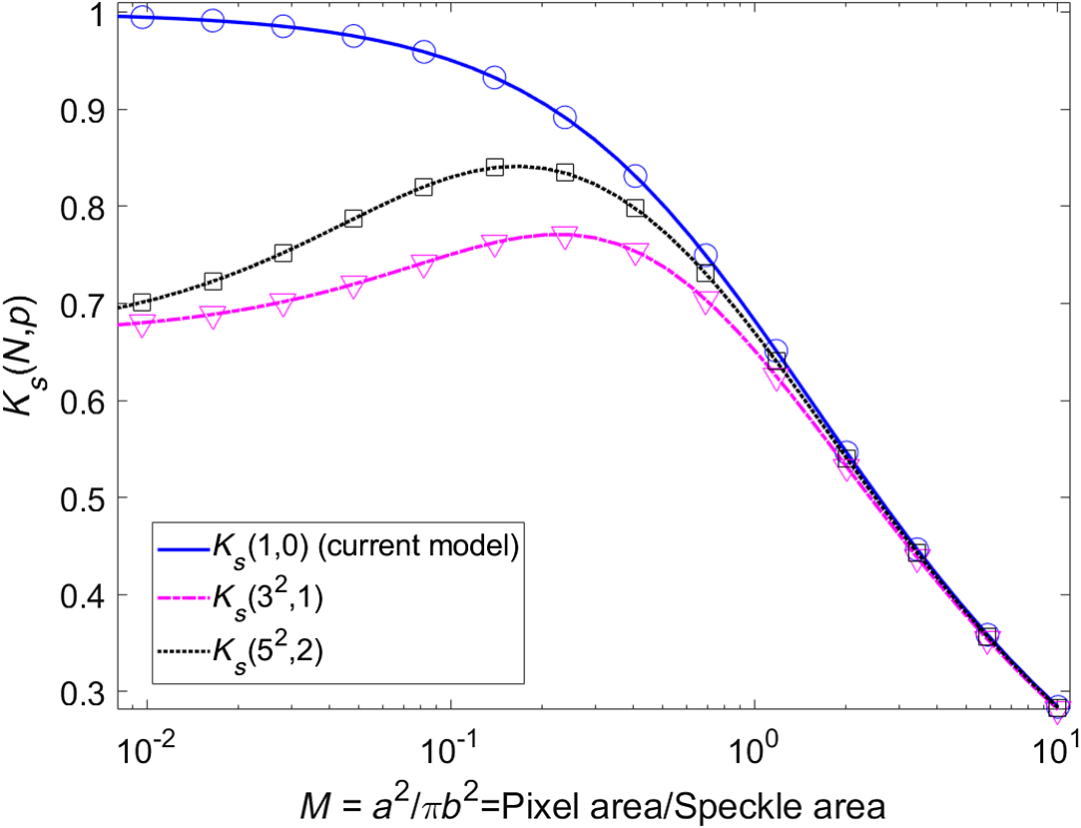
Solid lines show the contrast calculated using Eq. (6) for a correlation subregion of 1×1 (no spatial correlation), 3×3, and 5×5  pixels, in blue, pink, and black, respectively. The circle, triangle, and square marks show the numerical solution using Mathematica 11 for spatial correlation submatrices of 1×1, 3×3, and 5×5  pixels. Good agreement is observed between the numerical and analytical expressions.

Figure 3 shows the plots of the analytical (solid lines) and numerical (symbols) contrast for a correlation subregion of 1×1 (i.e., no spatial correlation), 3×3, and 5×5 pixels using Eq. (6). When the speckle size is larger than the pixel size (i.e., M<1), each speckle grain spans multiple pixels, resulting in spatial correlation among adjacent pixels within the sliding window. Due to this correlation, the standard deviation σ of intensity values within the sliding window decreases for M<1, resulting in an associated decrease in K. When the speckle size is smaller than the pixel size (i.e., M>1), each pixel contains intensity contributions from multiple speckle grains, leading to a lack of spatial correlation among adjacent pixels within the sliding window. In addition, the integration of multiple grains per pixel leads to a reduction in intensity variance in the speckle pattern.

In Fig. 3, it is show that for each size of sliding window, there is a different maximum contrast, hence, in order to maximize the contrast range, it is possible to choose the sliding window size for a given pixel and speckle area and vice versa, it is possible to choose the ratio M for a given sliding window size.

## 3 Experimental Validation of Proposed Model

**Fig. 4:**
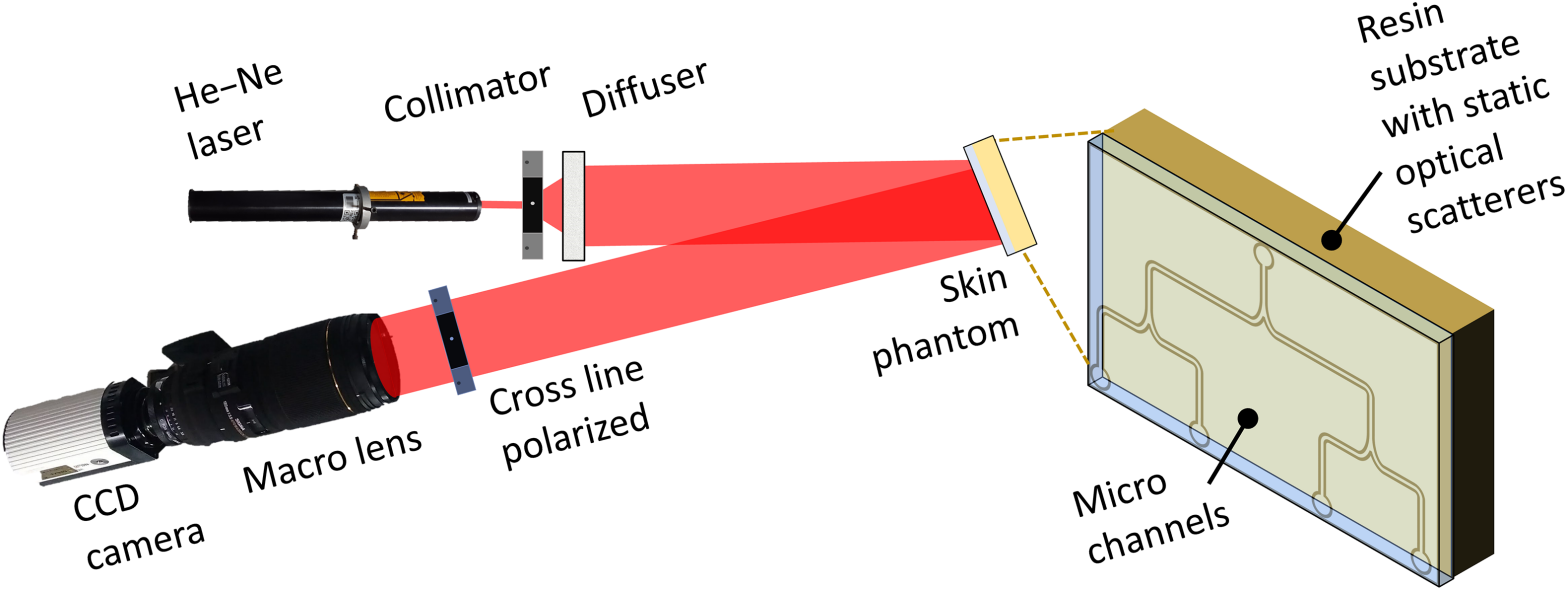
Experimental setup used to test the spatial speckle correlation model. An expanded and collimated 532 nm laser illuminated the skin phantom. A Retiga CCD camera equipped with a variable-aperture macroacquired speckle images. A linear polarizer was placed in front of the lens to mitigate specular reflectance contributions to the images.

Laser light from a coherent source (Verdi, Coherent Inc., λ=532  nm) was expanded and collimated. Then the light passed through a diffuser to achieve a homogeneous illumination of a flow phantom, which consisted of a microfluidic slide (thinXXS Microtechnology AG) with channel diameter of 300  μm. Intralipid (1%) was delivered with a syringe-based infusion pump into the channels at flow speeds from 4 to 20  mm/s in step of 2  mm/s. Raw speckle were recorded with a CCD camera (Retiga 2000R) (7.4  μm×7.4  μm pixel area). The camera was equipped with a macrolens with a variable aperture (Fig. 4) (see Ref. 38 for a detailed explanation of the experimental setup).

We modified the speckle size by changing the f# of the lens attached to the CCD camera:

speckle size=2.44(Mag+1)λf#, (12)

where Mag is the magnification of the lens. The spatial speckle contrast was calculated from the experimental data using a sliding window of $N=3^{2},7^{2}$, and $15^{2}$.

**Fig. 5:**
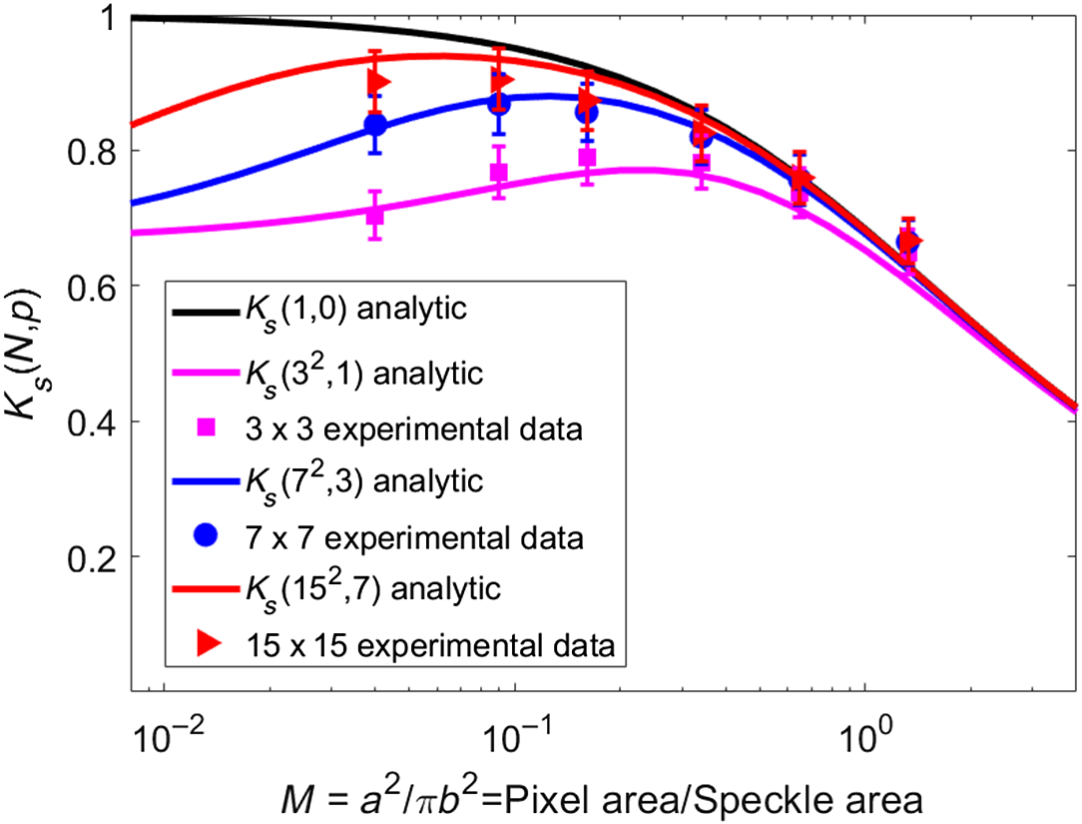
Solid lines show the contrast $K_{S}(3^{2},1)$, $K_{S}(7^{2},3)$, and $K_{S}(15^{2},)$ calculated using Eqs. (6) and (10) for spatial correlation submatrix of 3×3, 7×7, and 15×15  pixels in pink, blue, and red, respectively. Dots show the experimental data for 3×3, 7×7, and 15×15  pixels, in pink, blue, and red, respectively.

Figure 5 shows the mathematical prediction of speckle contrast using Eq. (6) and the experimental data. The curves generated with Eq. (7) overlap when M≥1 but diverge when M<1. Considering no correlation between neighboring pixels, the speckle contrast $K_{0}$ calculated by the model approaches 1 when M approaches zero (black continuous line) which is a contradiction, as the standard deviation should approach zero in that scenario and therefore the speckle contrast approaches zero, as observed experimentally. The speckle contrast must be <1 when M is <1. When correlation subregions (i.e., 3×3 and 5×5) are considered, speckle contrast reduces for lower values of M as expected.

The relationship between the pixel and the speckle grain size has an important effect on the speckle contrast.^39^ Figure 5 shows spatial contrast calculated using a standard spatial contrast algorithm ($K_{s}(1,0)$), which does not consider the potential spatial correlation of adjacent pixels, and calculations with our analytic model considering a sliding window of 3×3, 7×7, and 15×15  pixels. For different window sizes, the curves have a different maximum contrast at different values of M. Therefore, with our model, knowledge of M is sufficient to choose the appropriate sliding window and correlation subregion sizes to achieve the maximum speckle contrast.

We note that the experimental data in Fig. 5 agrees with calculations using our analytic model for all interrogated values of M. For M≪1 (the speckle grain size is larger than the pixel), choosing a submatrix at least as large as the speckle size to minimize loss of potential correlation between pixels is recommended.

Given a particular experimental setup and a sliding window size, M, N, and p are defined. Therefore, the correction factor $\beta =K_{s}(N,p)$ [Eq. (6)] should be utilized to determine the perfusion index ($\mathrm{PI}=1/\tau_{c}$) from the contrast (K) obtained with the sliding window, and given by Eq. (2) or Eq. (4). Considering $T\gg \tau_{c}$, the PI is given by:^34^ $\mathrm{PI}=\beta /TK^{2}$.

In Fig. 5, we use these window sizes merely as an example. However, the size of the sliding window is at the user’s discretion based on the characteristics of the speckle image they are analyzing. It should be noted that the larger the sliding window size is, the greater the loss of spatial resolution is.

**Fig. 6:**
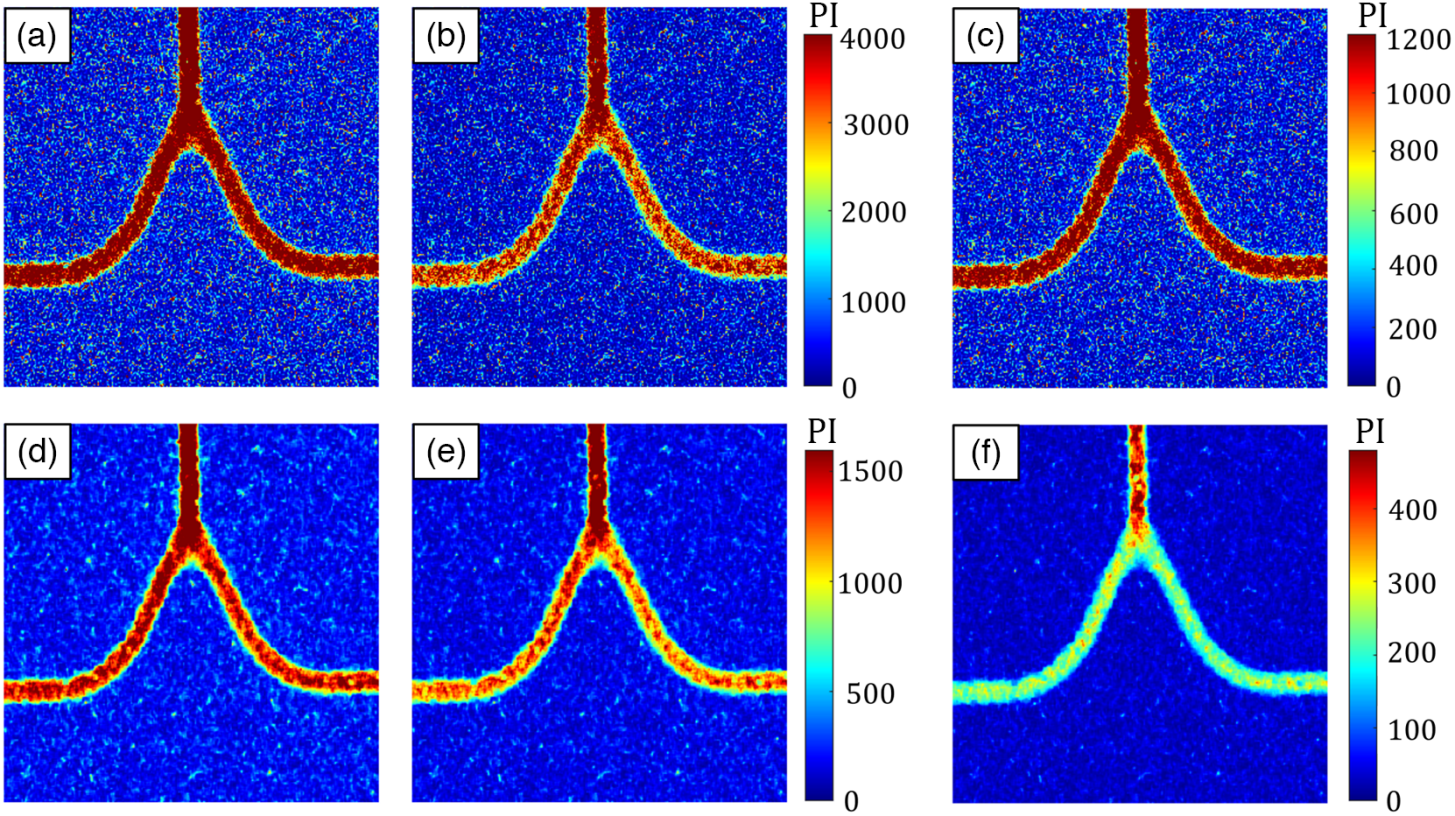
Accounting for the spatial correlation among pixels is required to calculate an accurate PI. (a)–(c) The $\mathrm{PI}=\beta /TK^{2}$ calculated using a sliding window of 3×3. (d)–(f) A sliding window of 7×7 pixel, with M=0.0338. (a), (d) The PI without considering the correlation between pixels, i.e., $\beta =K_{s}(1,0)=0.9827$. (b), (e) The corrected PI, $\beta =K_{s}(3^{2},1)=0.7073$ and $\beta =K_{s}(7^{2},3)=0.8198$, respectively. (c), (f) The difference between the first two columns.

Figure 6 illustrates the impact of the correction factor $\beta =K_{s}(N,p)$ on the PI image (for $T\gg \tau_{c}$), for two sliding window sizes (first row: 3×3 and second row: 7×7  pixels) and a fixed value of M=0.0338. The first column, (a) and (d), shows the PI without considering the correlation between pixels [Eq. (5)], resulting in $\beta =K_{s}(1,0)=0.9827$. The second column, (b) and (e), shows the corrected PI [Eq. (6)] by considering all the potential correlations within the sliding window. In this case, the correction factors are $K_{s}(3^{2},1)=0.7073$ and $K_{s}(7^{2},3)=0.8198$, respectively. The third column, (c) and (f), is the difference between the first two columns. As shown, when $N=3^{2}$, the error in PI calculation is nearly 30% and when $N=7^{2}$, the error is ∼20% with M=0.0338. The PI images shown in Fig. 6 represent the average PI over 30 frames.

From Fig. 6, we can observe that the difference between the first two columns is only the scalar multiplication by the correction factor, thus preventing the overestimation of the PI.

## 4 Conclusions

We present a more accurate estimation of the constant β and therefore of the contrast speckle contrast value by considering the effects of spatial correlation among the neighboring pixels. For a wide range of ratios of speckle grain size to pixel size, our model estimates the theoretical value of contrast more accurately than the standard model that does not consider the potential spatial correlation among adjacent pixels.^35^ For the special case of Gaussian illumination, we present an analytical solution for the spatial speckle contrast of any spatial correlation submatrix size. The resultant impact of our new model is a more accurate estimation of speckle contrast, which in turn should lead to improved estimations of tissue blood flow.

## 5 Appendix

The integrated intensity $I_{\eta ,\xi }$ over the physical area $a^{2}$ of the pixel (η,ξ) is given by

$$I_{\eta ,\xi }=\frac{1}{a^{2}}\int_{\text{pixel }\eta ,\xi }I(\overset{\to }{r})d^{2}\overset{\to }{r},$$ (13)

The mean intensity over the sliding window is

$$i=\frac{1}{N}\underset{\eta ,\xi }{\sum }I_{\eta ,\xi },$$ (14)

and the corresponding unbiased variance is

$$c=\frac{1}{N-1}\underset{\eta ,\xi }{\sum }{\left(I_{\eta ,\xi }-i\right)}^{2}.$$ (15)

The expected value of the variance is

$$\langle c\rangle =\frac{1}{N-1}(-\langle i^{2}\rangle N+\underset{\eta ,\xi }{\sum }\langle I_{\eta ,\xi }^{2}\rangle ).$$ (16)

where ⟨ ⟩ denotes the ensemble average.

Given two pixels within the sliding window with coordinates $\left(\eta^{\prime },\xi^{\prime }\right)$ and $\left(\eta^{\prime \prime },\xi^{\prime \prime }\right)$, respectively, the parameter $1/\mu_{\eta^{\prime \prime }-\eta \prime ,\xi^{\prime \prime }-\xi^{\prime }}$ depends on the spatial correlation between the given pixels and M the ratio between the pixel and speckle spot size. Due to it is not important, the actual pixel coordinates $\left(\eta^{\prime },\xi^{\prime }\right)$ and $\left(\eta^{\prime \prime },\xi^{\prime \prime }\right)$ but the distance between them $\eta =\eta^{\prime \prime }-\eta^{\prime }$ and $\xi =\xi^{\prime \prime }-\xi^{\prime }$. Therefore, the correlation factor $1/\mu_{\eta ,\xi }$ of the pixels (0,0) and (η,ξ) it is identical to $1/\mu_{\eta^{\prime \prime }-\eta^{\prime },\xi^{\prime \prime }-\xi^{\prime }}$. The correlation factor is given by the following expression:

$$\frac{1}{\mu_{\eta ,\xi }}=\frac{1}{a^{4}}\int_{\text{pixel }0,0}\int_{\text{pixel }\eta ,\xi }(g_{2}(\overset{\to }{r}-{\overset{\to }{r}}^{\prime })-1)d^{2}{\overset{\to }{r}}^{\prime d^{2}\overset{\to }{r}}=\frac{1}{a^{4}}\int_{\text{pixel }0,0}\int_{\text{pixel }\eta ,\xi }g_{1}^{2}(\overset{\to }{r}-{\overset{\to }{r}}^{\prime })d^{2}{\overset{\to }{r}}^{\prime d^{2}\overset{\to }{r}},$$ (17)

using Eqs. (13) and (17), we can rewrite $\left\langle I_{0,0}I_{\eta ,\xi }\right\rangle$ as

$$\langle I_{0,0}I_{\eta ,\xi }\rangle ={\left\langle I\right\rangle }^{2}(1+\frac{1}{\mu_{\eta ,\xi }}).$$ (18)

Computing $\left\langle i^{2}\right\rangle$ from Eq. (14), where ⟨ ⟩ denotes the ensemble average, and the summation spans all pixels within the sliding window of $\sqrt{N}\times \sqrt{N}$ pixels. Using the following notation:

$$\langle i^{2}\rangle =\frac{1}{N^{2}}\overset{\sqrt{N}}{\underset{\eta =-\sqrt{N}}{\sum }}\overset{\sqrt{N}}{\underset{\xi =-\sqrt{N}}{\sum }}\overset{\sqrt{N}}{\underset{\eta^{\prime \prime \prime }=-\sqrt{N}}{\sum }}\overset{\sqrt{N}}{\underset{\xi^{\prime \prime \prime }=-\sqrt{N}}{\sum }}\langle I_{0,0},I_{\eta ,\xi }\rangle .$$ (19)

It is possible to separate the summation into two sets of pixels (1) CW, where (η,ξ)∈(2p+1)×(2p+1) and (2) OW, where (η,ξ)∉(2p+1)×(2p+1).

For set CW, we focus on particular cases. When η=ξ=0, we call this a central correlation (see Fig. 3). When η=ξ≠0, we call this a diagonal correlation. When η≠ξ=0 or ξ≠η=0, we call this a lateral correlation. Finally, when (η,ξ)∈(2p+1)×(2p+1) and are not central, diagonal, or lateral correlations, we call these knight correlations due to the resemblance of the shape to the knight’s movement in chess. The other correlation, when the index (η,ξ)∈OW, is referred to as outsiders. Then Eq. (19) may rewritten as the sum of each type of correlations, i.e.:

$$\langle i^{2}\rangle =\frac{1}{N^{2}}(\underset{(\eta ,\xi )\in \text{central}}{\sum }\langle I_{0,0}I_{\eta ,\xi }\rangle +\underset{(\eta ,\xi )\in \text{diagonal}}{\sum }\langle I_{0,0}I_{\eta ,\xi }\rangle +\underset{(\eta ,\xi )\in \text{lateral}}{\sum }\langle I_{0,0}I_{\eta ,\xi }\rangle \;+\underset{(\eta ,\xi )\in \text{knight}}{\sum }\langle I_{0,0}I_{\eta ,\xi }\rangle +\underset{(\eta ,\xi )\in \text{outsiders}}{\sum }\langle I_{0,0}I_{\eta ,\xi }\rangle ).$$ (20)

For p=0, only the central correlation exists; hence, only the first summation in Eq. (20) is nonzero. Due to the symmetry in the correlation, some factors $1/\mu_{\eta ,\xi }$ are equivalent. For example, $1/\mu_{\eta ,\xi }$ is equivalent to $1/\mu_{\xi ,\eta }$, as well as any paired combination of (±η,±ξ). Approximately only the half of the pixels in one quadrant of the correlation subwindow are not equivalent to each other, without loss of generality, the fist quadrant is considered to classified/study these correlations, as it may see in Fig. 1(b). In this work, it has been classified the four different types of correlations: central, lateral, diagonal and knight.

For this reason, it is unnecessary to compute the correlation of all pixels. Instead, it is sufficient to compute the correlation of the pixels that are not equivalent and to weight their contributions appropriately.

Identifying the existence of N central correlations, one for each pixel in the correlation subregion, is straightforward. The other types of correlation are not so straightforward, but it can be demonstrated that for the diagonal correlation type, η=ξ≥1, there are $4{\left(\sqrt{N}-\eta \right)}^{2}$ equivalent correlations. For lateral type, η>ξ=0, there are $4\sqrt{N}(\sqrt{N}-\eta )$ equivalent correlations. For the knight type, 1≤ξ≠η≥2, there are $8(\sqrt{N}-\eta )(\sqrt{N}-\xi )$ equivalent correlations and let B(p) the number of outsider correlations.

For p=0, the correlation subwindow is only 1×1  pixel, i.e., only the autocorrelation is considered (central correlations), and Eq. (20) reduces to $\left\langle i^{2}\rangle =\frac{1}{N^{2}}N\langle I_{0,0}I_{0,0}\right\rangle$. For p=1, the correlation sub-window is 3×3  pixels, with only one central, one diagonal, and one lateral correlation. Therefore, Eq. (20) reduces to $\left\langle i^{2}\rangle =\frac{1}{N^{2}}(N\langle I_{0,0}I_{0,0}\rangle +4{\left(\sqrt{N}-1\right)}^{2}\langle I_{0,0}I_{1,1}\rangle +4\sqrt{N}(\sqrt{N}-1)\langle I_{0,0}I_{1,0}\rangle \right)$, which is the exact case analyzed by Skipetrov et al.^33^ The 1×1 pixels correlations subwindow (p=0) only has central correlation type, 3×3 is the first subwindow (p=1) with lateral and diagonal correlations type, and the 5×5 pixels correlation subwindow (p=2) is the first one that has one knight-type correlation, Eq. (20) reduce to

$$\langle i^{2}\rangle =\frac{1}{N^{2}}(N\langle I_{0,0}I_{0,0}\rangle +4({\left(\sqrt{N}-1\right)}^{2}\langle I_{0,0}I_{1,1}\rangle +{\left(\sqrt{N}-2\right)}^{2}\langle I_{0,0}I_{2,2}\rangle )+4\sqrt{N}((\sqrt{N}-1)\langle I_{0,0}I_{1,0}\rangle +(\sqrt{N}-2)\langle I_{0,0}I_{2,0}\rangle )+8(\sqrt{N}-2)(\sqrt{N}-1)\langle I_{0,0}I_{2,1}\rangle ).$$ (21)

Coyotl-Ocelotl analyzed this case.^37^

Henceforth p≥2, the first subregion with all types of correlation (diagonal, lateral, and knight) to avoid potential problems with missing terms in the summation. Then Eq. (20) is rewritten to

$$\langle i^{2}\rangle =\frac{1}{N^{2}}(N\langle I_{0,0}I_{0,0}\rangle +\overset{p}{\underset{\eta =\xi =1}{\sum }}4{\left(\sqrt{N}-\eta \right)}^{2}\langle I_{0,0}I_{\eta ,\eta }\rangle +\overset{p}{\underset{\eta =1}{\sum }}4\sqrt{N}(\sqrt{N}-\eta )\langle I_{0,0}I_{\eta ,0}\rangle \;+\overset{p-1}{\underset{\xi =1}{\sum }}\overset{p}{\underset{\eta =\xi +1}{\sum }}8(\sqrt{N}-\eta )(\sqrt{N}-\xi )\langle I_{0,0}I_{\eta ,\xi }\rangle +B(p)\langle I_{0,0}I_{\eta ,\xi }\rangle ).$$ (22)

Another possible correlation is what we call outsider correlations, which expand beyond the total number of central, diagonal, lateral, and knight correlations within the correlation subregion (2p+1)×(2p+1):

$$B(p)=N^{2}-(N+\overset{p}{\underset{\eta =\xi =1}{\sum }}4{\left(\sqrt{N}-\eta \right)}^{2}+\overset{p}{\underset{\eta =1}{\sum }}4\sqrt{N}(\sqrt{N}-\eta )+\overset{p-1}{\underset{\xi =1}{\sum }}\overset{p}{\underset{\eta =\xi +1}{\sum }}8(\sqrt{N}-\eta )(\sqrt{N}-\xi )).$$ (23)

Solving the summations, we obtain

$$B(p)=N^{2}-(N+(\frac{2p}{3}-4\sqrt{N}p+4\mathrm{Np}+2p^{2}-4\sqrt{N}p^{2}+\frac{4p^{3}}{3})+(-2\sqrt{N}p+4\mathrm{Np}-2\sqrt{N}p^{2})\;+(-\frac{2p}{3}+4\sqrt{N}p-4\mathrm{Np}-p^{2}+4Np^{2}+\frac{2p^{3}}{3}-4\sqrt{N}p^{3}+p^{4})).$$ (24)

Simplifying Eq. (24), we obtain

$$B(p)=N^{2}-{\left(p(1+p)-\sqrt{N}(1+2p)\right)}^{2}.$$ (25)

Here we assume that the outsider correlations are negligible by assuming that spatial correlations beyond the correlation subregion size are zero. For those pixels, $\langle I_{0,0},I_{\eta ,\xi }\rangle =\langle I_{0,0}\rangle \langle I_{\eta ,\xi }\rangle ={\left\langle I\right\rangle }^{2}$, and using Eq. (18), we obtain

$$\langle i^{2}\rangle =\frac{1}{N^{2}}(N{\left\langle I\right\rangle }^{2}(1+\frac{1}{\mu_{0,0}})+\overset{p}{\underset{\eta =1}{\sum }}4{\left(\sqrt{N}-\eta \right)}^{2}{\left\langle I\right\rangle }^{2}(1+\frac{1}{\mu_{\eta ,\eta }})+\overset{p}{\underset{\eta =1}{\sum }}4\sqrt{N}(\sqrt{N}-\eta ){\left\langle I\right\rangle }^{2}(1+\frac{1}{\mu_{\eta ,0}})\;+\overset{p-1}{\underset{\xi =1}{\sum }}\overset{p}{\underset{\eta =\xi +1}{\sum }}8(\sqrt{N}-\eta )(\sqrt{N}-\xi ){\left\langle I\right\rangle }^{2}(1+\frac{1}{\mu_{\eta ,\xi }})+B(p){\left\langle I\right\rangle }^{2}),$$ (26)

factorizing ${\left\langle I\right\rangle }^{2}$ and regrouping

$$\langle i^{2}\rangle =\frac{{\left\langle I\right\rangle }^{2}}{N^{2}}((N\frac{1}{\mu_{0,0}}+\overset{p}{\underset{\eta =1}{\sum }}4{\left(\sqrt{N}-\eta \right)}^{2}\frac{1}{\mu_{\eta ,\eta }}+\overset{p}{\underset{\eta =1}{\sum }}4\sqrt{N}(\sqrt{N}-\eta )\frac{1}{\mu_{\eta ,0}}\;+\overset{p-1}{\underset{\xi =1}{\sum }}\overset{p}{\underset{\eta =\xi +1}{\sum }}8(\sqrt{N}-\eta )(\sqrt{N}-\xi )\frac{1}{\mu_{\eta ,\xi }})\;+(N+\overset{p}{\underset{\eta =1}{\sum }}4{\left(\sqrt{N}-\eta \right)}^{2}+\overset{p}{\underset{\eta =1}{\sum }}4\sqrt{N}(\sqrt{N}-\eta )\;+\overset{p-1}{\underset{\xi =1}{\sum }}\overset{p}{\underset{\eta =\xi +1}{\sum }}8(\sqrt{N}-\eta )(\sqrt{N}-\xi ))+B(p)).$$ (27)

Using Eq. (23), some summation terms are eliminated with B(p), and Eq. (27) becomes

$$\langle i^{2}\rangle =\frac{{\left\langle I\right\rangle }^{2}}{N^{2}}((N\frac{1}{\mu_{0,0}}+\overset{p}{\underset{\eta =1}{\sum }}4{\left(\sqrt{N}-\eta \right)}^{2}\frac{1}{\mu_{\eta ,\eta }}+\overset{p}{\underset{\eta =1}{\sum }}4\sqrt{N}(\sqrt{N}-\eta )\frac{1}{\mu_{\eta ,0}}\;+\overset{p-1}{\underset{\xi =1}{\sum }}\overset{p}{\underset{\eta =\xi +1}{\sum }}8(\sqrt{N}-\eta )(\sqrt{N}-\xi )\frac{1}{\mu_{\eta ,\xi }})+N^{2}).$$ (28)

Similarly, to compute ⟨c⟩ [Eq. (16)], the term $\left\langle I_{\eta ,\xi }^{2}\rangle =\langle I_{\eta ,\xi }I_{\eta ,\xi }\right\rangle$ is equivalent to $\left\langle I_{\eta ,\xi }I_{\eta ,\xi }\rangle =\langle I_{0,0}I_{0,0}\right\rangle$. Using Eq. (18), Eq. (16) is rewritten as

$$\langle c\rangle =\frac{1}{N-1}(-\langle i^{2}\rangle N+\underset{\eta ,\xi }{\sum }{\left\langle I\right\rangle }^{2}(1+\frac{1}{\mu_{0,0}})),$$ (29)

solving the summation:

$$\langle c\rangle =\frac{1}{N-1}(-\langle i^{2}\rangle N+{\left\langle I\right\rangle }^{2}(1+\frac{1}{\mu_{0,0}})N),$$ (30)

substituting Eq. (28) and factorizing ${\left\langle I\right\rangle }^{2}$:

$$\langle c\rangle =\frac{{\left\langle I\right\rangle }^{2}}{N-1}(-\frac{1}{N^{2}}((N\frac{1}{\mu_{0,0}}+\overset{p}{\underset{\eta =1}{\sum }}4{\left(\sqrt{N}-\eta \right)}^{2}\frac{1}{\mu_{\eta ,\eta }}+\overset{p}{\underset{\eta =1}{\sum }}4\sqrt{N}(\sqrt{N}-\eta )\frac{1}{\mu_{\eta ,0}}\;+\overset{p-1}{\underset{\xi =1}{\sum }}\overset{p}{\underset{\eta =\xi +1}{\sum }}8(\sqrt{N}-\eta )(\sqrt{N}-\xi )\frac{1}{\mu_{\eta ,\xi }})+N^{2})N+(1+\frac{1}{\mu_{0,0}})N).$$ (31)

After simplification:

$$\langle c\rangle =\frac{{\left\langle I\right\rangle }^{2}}{N(N-1)}(N(N-1)\frac{1}{\mu_{0,0}}-\overset{p}{\underset{\eta =1}{\sum }}4{\left(\sqrt{N}-\eta \right)}^{2}\frac{1}{\mu_{\eta ,\eta }}-\overset{p}{\underset{\eta =1}{\sum }}4\sqrt{N}(\sqrt{N}-\eta )\frac{1}{\mu_{\eta ,0}}\;-\overset{p-1}{\underset{\xi =1}{\sum }}\overset{p}{\underset{\eta =\xi +1}{\sum }}8(\sqrt{N}-\eta )(\sqrt{N}-\xi )\frac{1}{\mu_{\eta ,\xi }}).$$ (32)

Finally, dividing ⟨c⟩ by ${\left\langle I\right\rangle }^{2}$ results in the calculation of the spatial contrast:

$$K_{s}^{2}(N,p)=\frac{1}{\mu_{0,0}}-\frac{1}{N(N-1)}(4\overset{p}{\underset{\eta =1}{\sum }}(\sqrt{N}-\eta )\sqrt{N}\frac{1}{\mu_{\eta ,0}}+4\overset{p}{\underset{\eta =1}{\sum }}{\left(\sqrt{N}-\eta \right)}^{2}\frac{1}{\mu_{\eta ,\eta }}\;+8\overset{p-1}{\underset{\xi =1}{\sum }}\overset{p}{\underset{\eta =\xi +1}{\sum }}(\sqrt{N}-\eta )(\sqrt{N}-\xi )\frac{1}{\mu_{\eta ,\xi }}).$$ (33)

Equation (33) is an analytic expression for speckle contrast that assumes both an arbitrary sliding window size and an arbitrary correlation subregion of pixels (p≥2). At this time, we do not consider any heterogeneity in illumination intensity $\left(g_{1}^{2}(\overset{\to }{r}-{\overset{\to }{r}}^{\prime })\right)$; this should be the subject of future work.

Equation (33) is expressed in terms of the factors $1/\mu_{\eta ,\xi }$. Those factors are given by Eq. (17), which may be simplified using the following changes of variables $w=x-x^{\prime }$, $\overset{¯}{w}=(x+x^{\prime })/2$, $u=y-y^{\prime }$, and $\overset{¯}{u}=(y+y^{\prime })/2$ equivalent to the triangular weight reported in the literature after Bandyopadhyay et al.^27^ Equation (17) can thus be rewritten as

$$\frac{1}{\mu_{\eta ,\xi }}=\frac{1}{a^{4}}\int_{-a(1+\xi )}^{-a\xi }(a(\xi +1)\{\int_{-a(1+\eta )}^{-a\eta }g_{1}^{2}(w,u)(a(\eta +1)+w)dw\;-\int_{-a\eta }^{a(1-\eta )}g_{1}^{2}(w,u)(a(\eta -1)+w)dw\}du\;-\int_{-a\xi }^{a(1-\xi )}(a(\xi -1)+u)\{\int_{-a(1+\eta )}^{-a\eta }g_{1}^{2}(w,u)(a(\eta +1)+w)dw\;-\int_{-a\eta }^{a(1-\eta )}g_{1}^{2}(w,u)(a(\eta -1)+w)dw\}du.$$ (34)

To this point, we have not made any assumptions about the shape of the correlation function $g_{1}^{2}(w,u)$. We now assume a Gaussian correlation shape:

$$g_{1}^{2}(w,u)={\left(\exp[-\frac{\pi }{2}\frac{\left(w^{2}+u^{2}\right)}{\pi b^{2}}]\right)}^{2}=\exp[-(w^{2}+u^{2})/b^{2}],$$ (35)

where $A_{c}$ is the correlation area and has been approximated to a circle of radius b.

Substituting Eq. (35) into Eq. (34), we arrive at the following expression for the factors $1/\mu_{\eta ,\xi }$, which is Eq. (10) in the main text:

$$\frac{1}{\mu_{\eta ,\xi }}=\frac{1}{4\pi^{2}M^{2}}\times (e^{-\pi M(\eta -1{)}^{2}}-2e^{-\pi M\eta^{2}}+e^{-\pi M(\eta +1{)}^{2}}\;+\pi \sqrt{M}((\eta -1)\mathrm{erf}[\sqrt{\pi M}(\eta -1)]-2\eta \;\mathrm{erf}[\sqrt{\pi M}\eta ]+(\eta +1)\mathrm{erf}[\sqrt{\pi M}(\eta +1)]))\;\times (e^{-\pi M(\xi -1{)}^{2}}-2e^{-\pi M\xi^{2}}+e^{-\pi M(\xi +1{)}^{2}}\;+\pi \sqrt{M}((\xi -1)\mathrm{erf}[\sqrt{\pi M}(-1)]-2\xi \mathrm{erf}(\sqrt{\pi M}\xi )+(\xi +1)\mathrm{erf}[\sqrt{\pi M}(\xi +1)])).$$ (36)

## Data Availability

Implementation of Eqs. (6) and (10) as functions in MATLAB and Mathematica are available at the GitHub repository: SpatialSpeckle, https://github.com/SpeckleContrast/SpatialSpeckle
